# The world should establish an early warning system for new viral infectious diseases by space‐weather monitoring

**DOI:** 10.1002/mco2.20

**Published:** 2020-07-23

**Authors:** Jiangwen Qu, N.Chandra Wickramasinghe

**Affiliations:** ^1^ Department of Infectious Disease Control Tianjin Centers for Disease Control and Prevention Tianjin China; ^2^ Buckingham Centre for Astrobiology University of Buckingham Buckingham UK; ^3^ Sri Lanka Centre for Astrobiology University of Ruhuna Sri Lanka; ^4^ National Institute of Fundamental Studies Kandy Sri Lanka; ^5^ Institute for the Study of Panspermia and Astroeconomics Gifu Japan

## Abstract

With the emergence of several new epidemics of viral infections – SARS, MERS, EBOLA, ZIKA, Influenza A (H1N1) pandemic,Covid‐2019 ‐ over the past 3 decades we suggest that a world‐wide programme of stratospheric surveillance and space weather monitoring should be urgently put in place without further delay.

## Graphical abstract

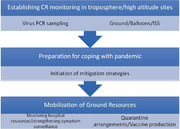

Since the 1950s, new infectious diseases have been emerging all over the planet, with serious repercussions on human health, global politics and the world economy. So far, more than 40 kinds of new infectious diseases have been found in the world, most of which are of viral origin.^1^ The occurrence of new viral infectious diseases often has the manifestation of strong contagion, rapid transmission, wide epidemic scope, high mortality. They are often difficult to predict or prevent, and can easily develop into serious public health emergencies causing grave international concern. However, despite all the advances in medical science and molecular biology, the scientific community still lacks sufficient understanding of the causes of the sudden emergence of new viral diseases, including relevant environmental and epidemiological factors that could be at work.

At the present time, our ability to detect new pathogens before they lead to epidemic or pandemic disease is still woefully inadequate. To quickly and effectively respond to emerging and unexplained infectious disease outbreaks a well thought‐out early‐warning technology system needs to be designed and put in place. The primary goal of such a system should be to guide us to discover new pathogens *before* they enter the human population. The recent COVID‐19 outbreak has confirmed the necessity of establishing such an early warning system.

There are a wide range of uncertain factors in the emergence and spread of new infectious diseases that needs to be explored. Within the current framework of medical knowledge most researchers think it impossible to predict when and where the next pandemic will occur. If one attempts to make any such prediction one needs to comprehensively detect countless potential pathogens in every possible location including the high atmosphere; but this method requires an extent of manpower and material resources that is not realistic at the present time. The most important prerequisite to early warning is to fully identify and explore those factors that are worth monitoring, and discover methods to detect an incipient epidemic in a local area before it begins to take on disastrous epidemic or pandemic proportions.

Once a potential pathogen is identified it is important next to determine through gene sequencing techniques (e.g. PCR) the precise species and its variants as quickly as possible. It would then be possible to launch a research program to produce effective preventive vaccines, devise treatment methods or manufacture drugs as may be relevant. If there is a way to make effective predictions of possible outbreaks of infectious diseases *ahead* of their occurrence we may have enough early warning so that actions to respond to an epidemic can be prompt and timely.

With a surge of new viral infectious diseases appearing recently we could ask the following questions: why do these new viral infectious diseases emerge at this particular time? Why do they manifest themselves in particular geographical areas? What are the factors that lead to the emergence of these new viral infectious diseases in the first place? How can we predict future new viruses? In order to attempt to answer these questions, we will need to engage in the exchange of information across diverse disciplines and embark on projects that involve inter‐disciplinary cooperation.

It is becoming amply clear that we cannot solve the most fundamental questions relating to the origin of new viral infectious diseases within the comfort zone of Earth‐bound medical science alone. First and foremost we need to abandon our tendency towards academic isolation and examine relevant facts from widely disparate fields ‐ physics, meteorology and space science – if we are to achieve our desired objectives. It is only with such an initiative that we can come anywhere near the prevention and control of infectious diseases in the long term.

At the present time we tend to believe that the emergence of new viral infectious diseases is mainly related to human activities or ecological factors. These include contact with wild animals, animal trade, new farmland reclamation, urbanization and rapid population growth, international tourism, population flow, climate change and similar factors. However, a stark fact that has either gone unnoticed or ignored is that most new viral infectious diseases seem to emerge in specific years ‐ immediately implying an astronomical context. The inevitable implication is that there must be some “unusual” cosmic factors in these specific years – in the Earth's configuration in relation to the wider Universe ‐ which play a decisive role. Therefore, only by discovering these “unusual” factors can we expect to find the root cause of occurrence of new viral infectious diseases, and thus be able to make scientific predictions of future new pandemics.

If particular years can be identified as a causative link an astronomical connection immediately suggests itself. The first lesson from astronomy is of course that the Earth is an insignificant planet in the universe – one of perhaps a hundred billion similar planets in our galaxy alone. As humans, we live on planet Earth but we also live in a far wider context – within a universe which is full of similar planets. Recent studies have shown the likelihood of life on Earth being part of a cosmic‐wide biosphere.^2^, ^3^ So it is by no means impossible that viruses which can relate to life on Earth may exist on a grand astronomical scale. We also know that the Earth is physically connected to the Universe – cosmic rays from the sun and from the galaxy reaches the Earth and also 10–100 of tonnes of meteoric dust (some of it organic) enters the Earth every single day. It is a pity that most modern researchers in the biological sciences do not realize these facts. It would appear very likely that all life on the Earth, including humans, will be genetically affected by the changes of our immediate space environment and various accompanying cosmic phenomena at any time. These include a variable flux of ionizing radiation – X rays, gamma rays, cosmic rays – that have a mutagenic effect on endemic terrestrial viruses, and even perhaps the addition of viruses from space, a wider cosmic environment, included in the daily tonnage of debris entering our planet from space.

Our previous studies have pointed out that sunspot extremes or +/‐1 year is an important risk factor for the occurrence of new viral infectious diseases.^4^, ^5^, ^6^, ^7^, ^8^ Sunspot numbers are related to solar activity. During solar maximum the sun produces high‐energy and low‐energy solar particles by a process of mass ejections from the sun's surface and solar flares. When the sun is least active, the solar magnetic field gets weaker and there will be more galactic cosmic rays entering the earth. We discussed in an earlier paper that the present time 2019–2020 is characterized by very low sunspot numbers and we are in a solar minimum that is the lowest in over 100 years. Ionizing radiation (cosmic rays) which peak at this time can cause virus mutation as well as genetic recombination, particularly if viruses are included in a biosphere extending even beyond the tropopause. In order to verify the correctness of this theory we issued an early warning in a letter to *Current Science* published in November 2019.^9^ Here we explicitly reminded the world that new infective viruses are likely to emerge in the ensuing months from mutations and/or ingress and that public health authorities must be vigilant and take necessary action. The emergence of the COVID‐19 outbreak uncannily confirms the accuracy of our early warning and highlights the urgency of taking appropriate precautions for future safety without further prevarication.

It is interesting in this context to recall that the word influenza comes from the Italian word *influencia*, meaning influence – and implying in this case influence of the stars. In the middle ages people saw connections between phenomena in the skies and events on Earth, and on this basis came to believe that many disasters were indeed related to the stars and celestial phenomena. To ancient people it seemed rational to believe that stars and the Universe could release “things” affecting the Earth! In certain respects, they appear to have been right.

As the philosopher Georg Wilhelm Friedrich once said, what a nation needs is for people to look up at the stars rather than down on the ground, and in this way a nation can have hope. Medical science should perhaps seek the cause of new viral infectious diseases by looking up at the stars. The famous Chinese book called “The Golden Mean” says: “Preparedness ensures success, and unpreparedness spells failure.” In our view “preparedness” at the present time involves setting up means to detect influences from outside the Earth, including conditions of space weather, cosmic ray flux, and arrival of novel cosmic strains of bacteria and viruses in the atmosphere. Following the discoveries of novel bacteria in the stratosphere at heights of 42km^10^, ^11^, ^12^ and the very recent discovery of bacteria on the exterior of the International Space Station orbiting at a height of 400km, the possibility of microbial life entering the Earth's surface should be considered seriously.^14^, ^15^ Furthermore, we should be guided by recent researches demonstrating that in the pristine environment of the Siera Nevada mountains (above the atmospheric boundary layer) there is a downward flux of viruses ranging in number from 0.26 × 10^9^to greater than 7 × 10^9^m^–2^ per day.^13^ They also confirmed that the deposition rates for bacteria were even greater, ranging from 0.3 × 10^7^to >8 × 10^7^m^–2^per day. To say that *all* this flux detected at a height of 4000km came from the ground is premature in our view.^15^

It is not too late to begin seriously planning for a space‐oriented monitoring strategy for detecting new viral/bacterial pathogens *before* they entered a human population. The present crisis relating to the emergence of COVID‐19 and its disastrous global spread^16^ is serious enough, but our preparation should include the possibility of more lethal pandemics in the future. Technologies for monitoring space weather, including cosmic ray flux and stratospheric microbial flux, do certainly exist at the present time. The desire and the will to deploy them for this purpose should be considered a prime responsibility of the nations of the world. Failing to do so will be a treachery to our intelligence.

We conclude by reiterating that a continual monitoring of the stratosphere for its bacterial and viral content as well as *in situ* gene sequencing which is well within our biotech and space capabilities should be carried out as a priority and without further delay. In the event of a new virus or a mutated or recombined virus being detected at, say, an altitude of say 40 km, the settling time to ground level to reach the human population could be many months. This would be enough time for mitigation strategies to be put in place. As Arthur C. Clarke once said the dinosaurs became extinct because they did not have space‐guard programme. Let us hope that we humans in 2020 could be wiser.

Finally we propose the following framework as a strategy for the future amelioration of pandemic‐caused disasters (Figure 1):

1. Establishing a cosmic ray monitoring stations including in the troposphere, as high priority.
2. Concurrently setting up virus monitoring stations, with in situ Polymerase Chain Reaction (PCR) equipment, in high altitude locations, preferably including one sited above the atmospheric boundary layer.  Balloon borne samplers should also be considered, as well virus (PCR) samplers installed on the exterior of a space station.
3. In the event of potentially harmful variants of a virus or a new virus being identified by PCR analysis, ground strategies are triggered into action.
4. Ground strategies must include checking resources for monitoring spread of the virus in the human population, providing hospital services (including intensive care if relevant) and in a longer term manufacturing appropriate vaccines, strengthening early symptom surveillance to identify abnormal cases.

**FIGURE 1:**
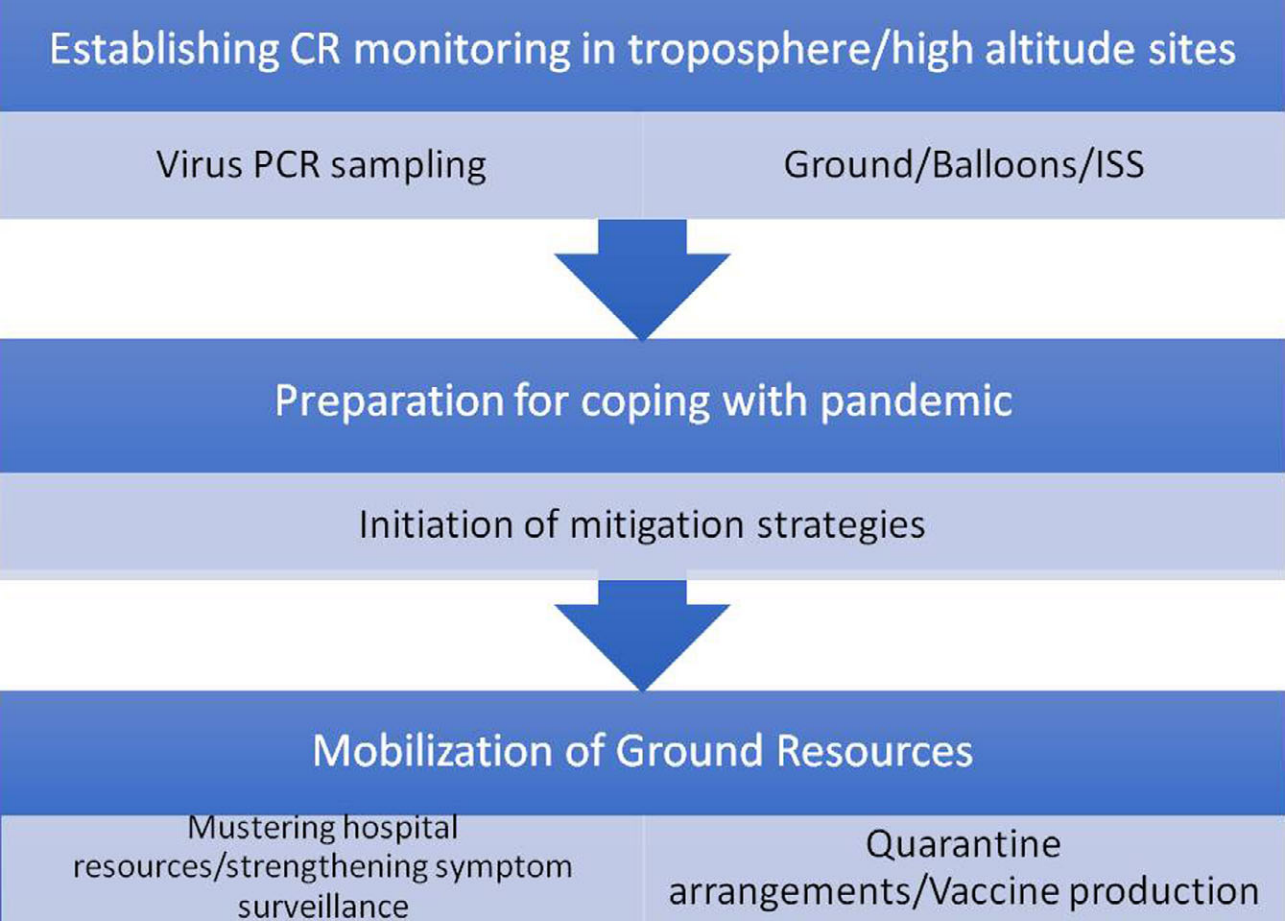
An early warning framework diagram based on space‐weather monitoring

## AUTHOR CONTRIBUTIONS

These authors contributed equally to this work.

## COMPETING INTERESTS

The authors have declared that no competing interests exist.
